# The effectiveness of problem-based learning and case-based learning teaching methods in clinical practical teaching in TACE treatment for hepatocellular carcinoma in China: a bayesian network meta-analysis

**DOI:** 10.1186/s12909-024-05615-8

**Published:** 2024-06-17

**Authors:** Jingxin Yan, Yonghao Wen, Xinlian Liu, Manjun Deng, Bin Ye, Ting Li, Huanwei Wang, Cui Jia, Jinsong Liao, Lushun Zhang

**Affiliations:** 1 West China Hospital, Sichuan University, Chengdu, China; 2 Department of Hepatopancreatobiliary Surgery, Affiliated Hospital of Qinghai University, Xining, China; 3 Department of Postgraduate, Qinghai University, Xining, China; 4 Department of Pathology and Pathophysiology, Chengdu Medical College, Chengdu, China; 5 Department of General Surgery, Rongxian People’s Hospital, Zigong, China; 6 Department of Orthopedics, Sichuan Provincial People’s Hospital, Chengdu, China; 7 Department of Ultrasonography, Hainan General Hospital/Hainan Affiliated Hospital of Hainan Medical University, Haikou, 570100 China; 8 Department of Anesthesiology, Affiliated Hospital of Chengdu University, Chengdu University, Chengdu, China

**Keywords:** Problem-based learning, Case-based learning, Transarterial chemoembolization, Bayesian method, Network meta-analysis

## Abstract

**Purpose:**

To investigate the effectiveness of problem-based learning (PBL) and case-based learning (CBL) teaching methods in clinical practical teaching in transarterial chemoembolization (TACE) treatment in China.

**Materials and methods:**

A comprehensive search of PubMed, the Chinese National Knowledge Infrastructure (CNKI) database, the Weipu database and the Wanfang database up to June 2023 was performed to collect studies that evaluate the effectiveness of problem-based learning and case-based learning teaching methods in clinical practical teaching in TACE treatment in China. Statistical analysis was performed by R software (4.2.1) calling JAGS software (4.3.1) in a Bayesian framework using the Markov chain-Monte Carlo method for direct and indirect comparisons. The R packages “gemtc”, “rjags”, “openxlsx”, and “ggplot2” were used for statistical analysis and data output.

**Results:**

Finally, 7 studies (five RCTs and two observational studies) were included in the meta-analysis. The combination of PBL and CBL showed more effectiveness in clinical thinking capacity, clinical practice capacity, knowledge understanding degree, literature reading ability, method satisfaction degree, learning efficiency, learning interest, practical skills examination scores and theoretical knowledge examination scores.

**Conclusions:**

Network meta-analysis revealed that the application of PBL combined with the CBL teaching mode in the teaching of liver cancer intervention therapy significantly improves the teaching effect and significantly improves the theoretical and surgical operations, meeting the requirements of clinical education.

**Supplementary Information:**

The online version contains supplementary material available at 10.1186/s12909-024-05615-8.

## Introduction

Hepatocellular carcinoma (HCC) is one of the leading causes of cancer-related death worldwide, and newly diagnosed cases increase annually [1]. More than 50% of newly diagnosed patients are reported in China, with an age-standardized incidence rate of 8.6 per 100,000 individuals annually [2]. China is a country with a high burden of hepatitis, which indicates that HCC is one of the main focuses of medical investment in China. According to Western and Eastern experts’ consensus and guidelines [3–5], transarterial chemoembolization (TACE), an interventional method that embolizes the tumor-feeding vascular with embolization materials and chemotherapy drugs, is considered the first choice for most patients with advanced-stage HCC, providing opportunities for surgery. In addition, clinical evidence has also confirmed the effectiveness of TACE and its related protocol in different clinical settings [6].

With the rapid development of medical education, therefore, cultivating excellent medical professionals is particularly crucial. In clinical education, traditional lecture-based teaching has shortcomings; for example, teachers place too much emphasis on knowledge and passive student learning [7], resulting in low learning efficiency, insufficient clinical thinking ability, and poor clinical practice ability for students. Moreover, the teaching of interventional radiology, including TACE and other related disciplines, is highly specialized, with relatively few class hours and relatively short internship times, making it difficult to master the outline knowledge in a short period of time. Therefore, to improve teaching effectiveness, it is necessary to break the constraints of traditional teaching methods and strive to find more effective teaching methods.

The problem-based learning (PBL) teaching method emphasizes students’ active learning as the main focus, rather than the traditional lecture-based teaching method. It is based on a student-centered education approach, guided by teachers and based on questions, to introduce relevant basic knowledge [8]. Through group discussions, students independently collect data and discover and solve problems, and this teaching model can cultivate students’ active learning and innovation. The case-based learning (CBL) teaching method is based on typical cases, using real cases from clinical work in teaching. Before the teacher systematically explains, students are asked to contact the patient themselves in advance and carefully inquire about their medical history and clinical examinations [9]. Then, relevant information is collected based on the patient’s specific situation (such as similar patient onset factors, diagnosis and treatment plans, treatment clinical reactions, and posttreatment effects). Finally, a preliminary treatment plan will be formed by students, and teachers will continuously improve treatment plans and apply relevant theoretical knowledge for analysis.

Regarding the use of PBL and CBL for TACE teaching, only several Chinese studies found that PBL and CBL could benefit the students and trainees, as TACE teaching requires mastery of various benign and malignant tumors of the liver, including atypical cases, and interspersed with different teaching contents. Besides, TACE is a discipline that requires not only solid theoretical knowledge, but also high mastery and proficiency in practical operational skills. Therefore, the requirements for teaching methods should also be increased [10].

Although some published randomized controlled trials and observational studies have examined CBL and PBL in clinical education in TACE, there is currently no consensus on the advantages or disadvantages of these two methods. With our study, We hope to provide the optimum educational method for TACE. Therefore, in this study, we conducted a high-quality Bayesian network meta-analysis and systematic review to explore the effectiveness of the PBL and CBL methods in the clinical practical teaching of TACE in China, with the aim of providing a new perspective for the clinical education of TACE.

## Methods

### Study design

In this study, the Bayesian network meta-analysis was performed following the Preferred Reporting Items for Systematic reviews and Meta-analyses statement [11]. We used a Bayesian network meta-analysis because of its superiority in accounting for the pooled effect and providing precise calculations for related data.

### Data sources and search

A comprehensive search of PubMed, Chinese National Knowledge Infrastructure database (CNKI), Weipu database and Wanfang database up to June 2023 was performed. Table S1 lists the search strategy, inclusion criteria, and exclusion criteria.

### Data extraction and risk of bias assessment

Two independent reviewers carried out the research and data extraction, and any disagreements were resolved by a third author. Data on study details (first author, study design, year of publication, study population and sample size.) and primary outcomes were extracted into an Excel sheet. We also extracted data on the performance of the difference teaching method. We used the methods of the Cochrane Handbook for Systematic Reviews of Interventions to assess the risk of the randomized controlled trials [12]. In addition, the Newcastle–Ottawa scale was adopted to evaluate observational studies [13].

### Data synthesis and statistical analysis

We conducted the network meta-analyses for theoretical knowledge examination scores, practical skills examination scores, and the questionnaire entry using a random-effect model in a Bayesian framework.

Statistical analysis was performed by R software (4.2.1) calling JAGS software (4.3.1) in a Bayesian framework using the Markov chain-Monte Carlo method for direct and indirect comparisons. The R packages “gemtc”, “rjags”, “openxlsx”, and “ggplot2” were used for statistical analysis and data output. Parameter settings: the number of chains was 6, the initial value was 2.5, the number of adaptation (or tuning) iterations was 50,000, the number of simulation iterations was 200,000, and the thinning factor was 10.

The network plot and funnel plot were drawn using Stata software (version 16).

Furthermore, statistical heterogeneity and inconsistency were evaluated using the Q test and the statistic inconsistency index (I^2^). An I^2^ value greater than 50% is generally considered to indicate a substantial level of heterogeneity, which consequently initiates sensitivity analysis to identify the source [14]. Discontinuous data in a Bayesian framework were calculated with the risk ratio (RR) and its 95% confidence interval (CI), and the natural logarithm of RR (LnRR) was used to estimate the outcomes. Continuous data in a Bayesian framework were calculated with the mean difference (MD) and its 95% CI. Accordingly, we performed a pairwise meta-analysis on comparisons on the basis of the frequentist approach to compare with the corresponding pooled results from the Bayesian framework. We used a line diagram to calculate the rank probability of different therapies, in which the X axis represents probability, while the Y axis represents ranking from first to last [15, 16].

## Results

### Study selection and characteristics of included studies

**Fig. 1:**
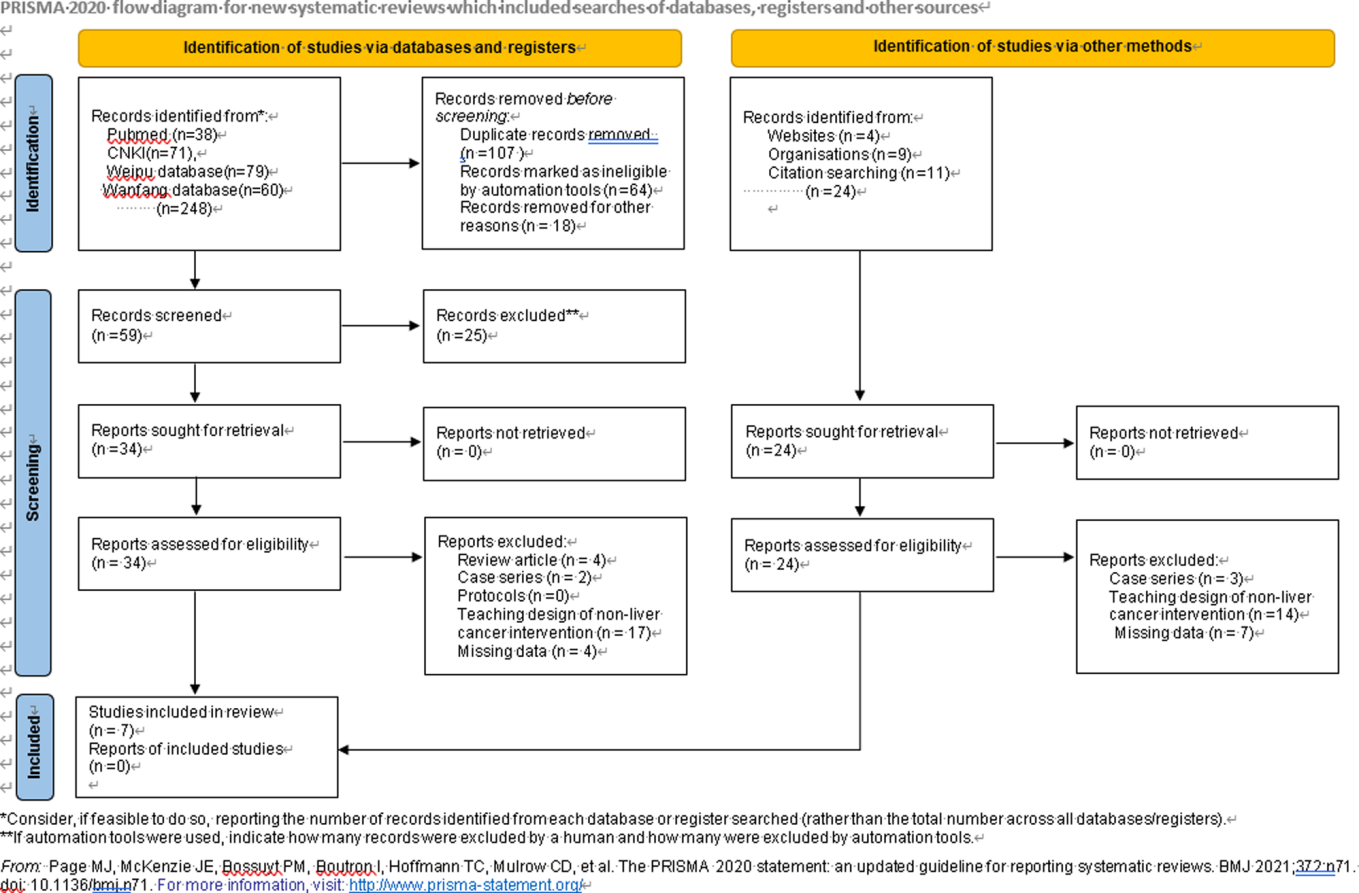
Flowchart of the literature search process

A preliminary search yielded 248 articles, of which 107 were duplicates. After removing duplicates by automated tools, we reviewed the abstracts of the remaining studies, and 134 articles did not meet the inclusion criteria. Finally, 7 studies (five RCTs [10, 17–20] and two observational studies [21, 22]) were included in the meta-analysis. Figure 1 shows the study selection flowchart of the literature search process.

Description of the selected studies: first author, year of publication, country, intervention, the most important results. In Table 1. The study quality of the included studies is shown in Tables 2 and 3.

**Table 1:** The characteristics of the included studies

Studies	Participants	Year	Country	Intervention	Outcomes
Wang ZC	Medical students	2020	China	PBL + CBL = 15 vs. CBL = 15 vs. PBL = 15	a, g, h, i
Wang YC	Medical students	2020	China	PBL + CBL = 28 vs. CBL = 28 vs. PBL = 28	a, b, c, e, f, g, h, i
Du MJ	Medical students	2018	China	PBL = 50 vs. LBL = 50	a, b
Wang LZ	Medical students	2015	China	PBL = 28 vs. LBL = 27	a, b
Sun Y	surgical trainees	2021	China	PBL + CBL = 24 vs. LBL = 24	a, b, c, d, f, i
Sha L	Medical students	2018	China	PBL + TBL = 43 vs. LBL = 43	a, b, c, d, g, i
Li LF	Medical students	2020	China	PBL = 47 vs. LBL = 47	a, b, e

PBL + CBL: Combined with Problem-Based Learning and Case-based learning, PBL: Problem-Based Learning, PBL + TBL: Combined with Problem-Based Learning and Team-based learning, CBL: Case-based learning, LBL: lecture-based learning, a: theoretical knowledge examination scores, b: practical skills examination scores, c: learning interest, d: learning efficiency, e: method satisfaction degree, f: literature reading ability, g: Knowledge understanding degree, h: clinical practice capacity, i: clinical thinking capacity

**Table 2:** Results of quality assessment using Cochrane Tool for RCTs

Studies	Random sequence generation	Allocation concealment	Blinding	Incomplete outcome	Selective report	Other bias
Wang ZC	Unclear	Unclear	High risk	Low risk	Low risk	Low risk
Wang YC	Unclear	Unclear	High risk	Low risk	Low risk	Low risk
Sun Y	Low risk	Unclear	High risk	Low risk	Low risk	Low risk
Li LF	Unclear	Unclear	High risk	Low risk	Unclear	Unclear

**Table 3:** Results of quality assessment using Newcastle–Ottawa scale for observational studies

Study selection	Representativeness of the exposed cohort	Selection of the nonexposed cohort	Ascertainment of exposure	Demonstration that outcome of interest was not present at start of study	Comparability of cohorts on the basis of the design or analysis	Assessment of outcome	follow-up long enough for outcomes to occur	Adequacy of follow-up of cohorts	Quality score
Wang LZ	1	1	1	1	1	0	1	1	7
Du MJ.	1	1	0	1	1	0	1	1	6
Sha L	1	1	1	1	1	0	1	1	7

### Findings of the bayesian network meta-analysis

#### Bayesian network meta-analysis of theoretical knowledge examination scores

**Fig. 2:**
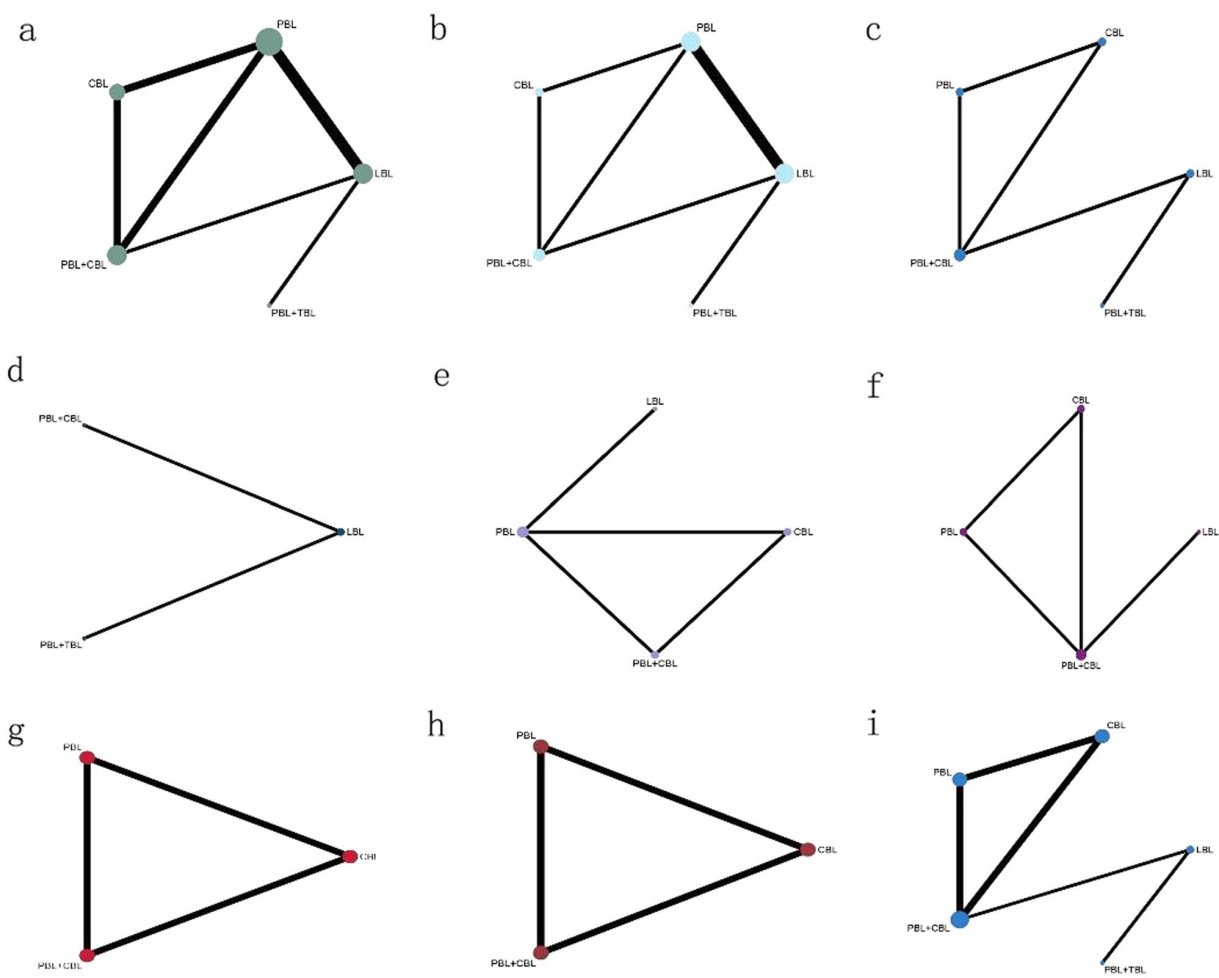
Network plot. (**A**) Theoretical knowledge examination scores; (**B**) practical skills examination scores; (**C**) learning interest; (**D**): learning efficiency; (**E**) method satisfaction degree; (**F**) literature reading ability; (**G**) knowledge understanding degree; (**H**) clinical practice capacity; (**I**) clinical thinking capacity

**Fig. 3:**
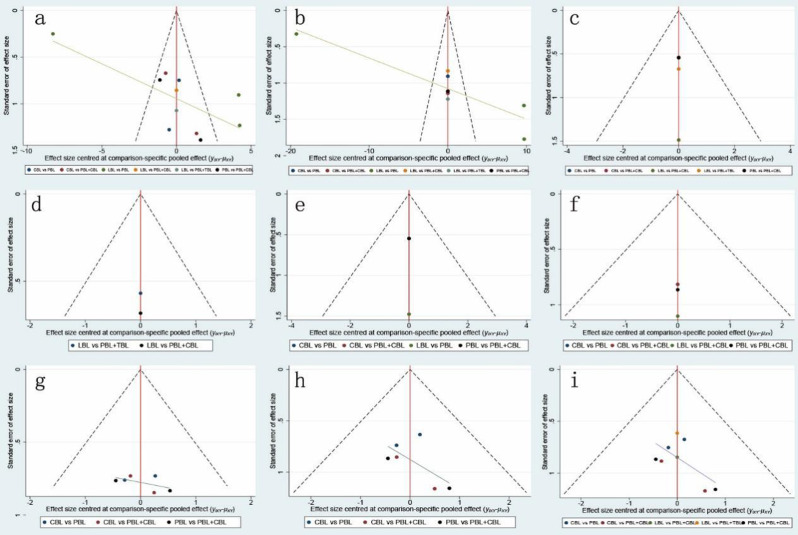
Funnel plot of outcomes. (**A**) Theoretical knowledge examination scores; (**B**) practical skills examination scores; (**C**) learning interest; (**D**): learning efficiency; (**E**) method satisfaction degree; (**F**) literature reading ability; (**G**) knowledge understanding degree; (**H**) clinical practice capacity; (**I**) clinical thinking capacity

Theoretical knowledge examination scores were reported in all studies. Eligible comparisons of outcomes are presented in the network plot (Fig. 2a). We used a table (Table S2) to describe the effect of 5 interventions on the theoretical knowledge examination scores in participants with a total of 6 comparisons with LnRR. No significant publication bias was found (Fig. 3a). PBL in combination with TBL showed the best improvement in the theoretical knowledge examination scores, followed by PBL in combination with CBL (Figure S1).

#### Bayesian network meta-analysis of practical skills examination scores

Practical skills examination scores were reported in 6 studies [10, 18–22]. Eligible comparisons of outcomes are presented in the network plot (Fig. 2b). We used a table (Table S3) to describe the effect of 5 interventions on the practical skills examination scores in participants with a total of 6 comparisons. No significant publication bias was found (Fig. 3b). PBL in combination with TBL showed the best improvement in the practical skills examination scores, followed by PBL (Figure S2).

#### Bayesian network meta-analysis of learning interest

Learning interest was reported in 3 studies [18, 20, 22]. Eligible comparisons of outcomes are presented in the network plot (Fig. 2c). We used a table (Table S4) to describe the effect of 5 interventions on learning interest in participants with a total of 5 comparisons. No significant publication bias was found (Fig. 3c). PBL in combination with TBL showed the best improvement in learning interest, followed by PBL in combination with CBL (Figure S3).

#### Bayesian network meta-analysis of learning efficiency

Learning efficiency was reported in 2 studies [20, 22]. Eligible comparisons of outcomes are presented in the network plot (Fig. 2d). We used a table (Table S5) to describe the effect of 3 interventions on learning efficiency in participants with a total of 2 comparisons. No significant publication bias was found (Fig. 3d). PBL in combination with TBL showed the best improvement in learning efficiency, followed by PBL in combination with CBL (Figure S4).

#### Bayesian network meta-analysis of method satisfaction degree

Method satisfaction degree were reported in 2 studies [10, 18]. Eligible comparisons of outcomes are presented in the network plot (Fig. 2e). We used a table (Table S6) to describe the effect of 4 interventions for the method satisfaction degree in participants with a total of 4 comparisons. No significant publication bias was found (Fig. 3e). PBL in combination with CBL is the most satisfied among students, followed by PBL (Figure S5).

#### Bayesian network meta-analysis of literature reading ability

Literature reading ability was reported in 2 studies [18, 20]. Eligible comparisons of outcomes are presented in the network plot (Fig. 2f). We used a (Table S7) to describe the effect of 4 interventions on the literature reading ability in participants with a total of 4 comparisons. No significant publication bias was found (Fig. 3f). PBL in combination with CBL showed the best improvement in literature reading ability, followed by PBL (Figure S6).

#### Bayesian network meta-analysis of knowledge understanding degree

Knowledge understanding degree were reported in 2 studies [17, 18]. Eligible comparisons of outcomes are presented in the network plot (Fig. 2g). We used a league matrix table (Table S8) to describe the effect of 3 interventions for the knowledge understanding degree in participants with a total of 3 comparisons. No significant publication bias was found (Fig. 3g). PBL in combination with CBL showed the best improvement in the knowledge understanding degree, followed by PBL (Figure S7).

#### Bayesian network meta-analysis of clinical practice capacity

Clinical practice capacity was reported in 2 studies [17, 18]. Eligible comparisons of outcomes are presented in the network plot (Fig. 2h). We used a (Table S9) to describe the effect of 3 interventions on the clinical practice capacity in participants with a total of 3 comparisons. No significant publication bias was found (Fig. 3h). PBL in combination with CBL showed the best improvement in the clinical practice capacity, followed by PBL (Figure S8).

#### Bayesian network meta-analysis of clinical thinking capacity

Clinical thinking capacity was reported in 4 studies [17, 18, 20, 22]. Eligible comparisons of outcomes are presented in the network plot (Fig. 2i). We used a table (Table S10) to describe the effect of 5 interventions on the clinical thinking capacity in participants with a total of 5 comparisons. No significant publication bias was found (Fig. 3i). PBL in combination with CBL showed the best improvement in the clinical thinking capacity, followed by CBL (Figure S9).

## Discussion

In this meta-analysis of randomized controlled trials and observational studies, we found that the combination of PBL and CBL is the most effective teaching method in TACE treatment in China. The combination of PBL and CBL showed more effectiveness in clinical thinking capacity, clinical practice capacity, knowledge understanding degree, literature reading ability, method satisfaction degree, learning efficiency, learning interest, practical skills examination scores and theoretical knowledge examination scores. In China, interventional therapy has been widely carried out since the 1980s [23], but the education method is still at an early stage. With this systematic review and meta-analysis, we summarized the current educational practice in China in terms of TACE.

To our knowledge, this is the first network evidence-based study investigating the effectiveness of different teaching methods of TACE in China. In addition, this is also the first systematic review and meta-analysis that has been carried out to investigate the interventional teaching method of TACE.

Since PBL was posted in the 1960s in response to dissatisfaction with traditional medical education, scholars have found that PBL can contribute to knowledge retention, student satisfaction, motivation, and critical thinking from many perspectives on teaching [24–28] In addition, PBL is currently widely used in North America and Asia, and PBL is considered a successful implementation of current medical education, but the utilization of PBL is different in different regions, showing no difference in geographical origin [29]. Even though some studies have been published, the heterogeneity within the method, region, individuals and outcomes left some difficulties for medical educational researchers. As a result, some studies showed inconsistent research results in the outcomes when PBL was used [30–32]. It should be noted that the current definition of CBL is not completely clear, and researchers from different countries have proposed definitions of CBL with different details but the same core [33]. CBL and PBL allow students to obtain and integrate clinical knowledge before their internship career. However, none of the studies mentioned above investigated interventional treatment teaching methods, so our meta-analysis provides value in this vacuum field.

With the rapid development of clinical medicine, traditional medical teaching methods cannot meet the needs of the medical education system. For instance, medical students who cannot master the content of anatomy classes solely through books and lecture teaching need to dissect cadavers to understand the structure of the human body. Similarly, they cannot master the methods and procedures of TACE solely through traditional education methods. There are some possible reasons that may explain why the combination of PBL and CBL showed a better effectiveness in TACE teaching in China. Unlike traditional teaching methods, the combination of PBL and CBL allows for more interaction between students and teachers, improving students’ perceptions of learning [34, 35]. In addition, the combination of PBL and CBL may inspire students to engage in theoretical knowledge learning and practical skills, forging a preliminary mind of clinical logic and a stronger grasp of experimental processes [36].

Recent research highlights the significance of problem-based learning (PBL) and case-based learning (CBL) in education. Studies show that PBL and CBL enhance students’ motivation, engagement, and knowledge construction. Furthermore, longitudinal analyses indicate that social learning dynamics within PBL groups contribute to learning outcomes [37]. Additionally, utilizing case-based learning has been shown to improve clinical reasoning skills in medical education [38]. Realist methods are also increasingly utilized in medical education research to gain deeper insights into learning processes [39]. These findings underscore the importance of incorporating PBL, CBL, and realist methodologies in educational practices.

The regional nature of the study results warrants consideration due to China’s distinct educational environment, cultural context, and medical system. China’s evolving educational landscape, influenced by cultural factors and a shift towards student-centered learning approaches, may impact the applicability of findings on problem-based learning (PBL) and case-based learning (CBL) effectiveness [40]. Additionally, variations in healthcare systems and medical education practices highlight the need for caution in generalizing results beyond China. Future research should explore the transferability of PBL and CBL to diverse international contexts, considering cultural and educational differences [41, 42].

We used Bayesian method to perform this network meta-analysis, as Bayesian method provides more accurate estimates for small samples because this method takes into account possible bias, reaching more accurate estimates for small samples [43]. After analyzing data through prior information, the resulting posterior information can be used again as prior information in the next statistical calculation process, especially in the process of clinical decision-making, which is more efficient and reliable [44, 45]. Besides, the parameter settings is chosen based on our previous studies, which reduce errors caused by insufficient iterations [46].

The inconsistences of the findings across individual studies should be noted. For example, all included studies did not adopt uniform outcome measures, as there is no standard examination to test the theoretical scores and practical skills. Hence, a standard examination should be established in the future. In this meta-analysis, we synthesized the results to assess the total effectiveness; however, these differences with the results may lead to significant heterogeneity.

Similar to any meta-analysis and evidence-based study, the limitations of this meta-analysis should be noted. First, we included both RCTs and observational studies in this meta-analysis, which will undoubtedly lead to bias in the results and conclusions. Second, some of the outcomes were evaluated subjectively, which may lead to inconsistent results among individuals. Third, as only seven studies were included in this meta-analysis, the sample size of the included studies was exceedingly small, which undoubtedly led to bias that affect the accuracy of the study. Fourth, all participants were from China, so researchers outside China should interpret our results with caution. Fifth, the inconsistencies within included studies might arise from subjective evaluation metrics.

## Conclusion

In conclusion, our study found that the combination of PBL and CBL in TACE teaching education was able to improve knowledge learning, practical skills and other important skills in teaching. However, due to the small sample size of the included individuals and the limitations within the study, further high-quality studies are needed to verify our results and conclusions.

### Electronic supplementary material

Below is the link to the electronic supplementary material.

Supplementary Material 1

Supplementary Material 2

## Data Availability

The datasets used and analyzed during this study are available from the corresponding author on reasonable request.

## Abbreviations

CNKI: Chinese National Knowledge Infrastructure
TACE: Transarterial chemoembolization
PBL: Problem-based Learning
CBL: Case-based Learning Teaching
HCC: Hepatocellular carcinoma
RR: Risk ratio
CI: Confidence interval
LnRR: Natural logarithm of RR
MD: Mean difference
