# Supplementary material for: The effectiveness of problem-based learning and case-based learning teaching methods in clinical practical teaching in TACE treatment for hepatocellular carcinoma in China: a bayesian network meta-analysis
Source: BMC Med Educ. 2024 Jun 17;24:665. doi: 10.1186/s12909-024-05615-8 (PMC11184776; doi:10.1186/s12909-024-05615-8)
Supplement: Supplementary file 1 — Supplementary Material 1 [file 12909_2024_5615_MOESM1_ESM.docx]

| **Table S1** | | |  |  |  |  |  |  |  |  |
| --- | --- | --- | --- | --- | --- | --- | --- | --- | --- | --- |
| Search strategy summary. | |  | | |  | | | | | |
| Search item | | | | Specification | | | |  |  |  |
| Time span | | |  | Jan 1, 1980 to June, 2023 | | |  |  |  |  |
| Databases and other sources searched | | | | PubMed, Chinese National Knowledge Infrastructure database (CNKI), Weipu database and Wanfang database | | | | |  |  |
| Search terms used | | | | #1 hepatocellular carcinoma [Mesh Terms] | | | | |  |  |
|  |  | |  | #2 unresectable hepatocellular carcinoma [Title/ Abstract] | | | | | |  |
|  |  | |  | #3 advanced hepatocellular carcinoma [Title/Abstract] | | | | | |  |
|  |  | |  | #4 primary liver cancer [Title/Abstract] | | | |  |  |  |
|  |  | |  | #5 #1 OR #2 OR #3 OR #4 | | |  |  |  |  |
|  |  | |  | #6 TACE [Title/Abstract] | | |  |  |  |  |
|  |  | |  | #7 Transarterial chemoembolization [Mesh Terms] | | | | |  |  |
|  |  | |  | #8 #6 OR #7 | |  |  |  |  |  |
|  |  | |  | #9 Hepatic artery infusion chemotherapy [Title/Abstract] | | | | | |  |
|  |  | |  | #10 HAIC [Title/Abstract] | | |  |  |  |  |
|  |  | |  | #11 #9 OR #10 | |  |  |  |  |  |
|  |  | |  | #12 Problem-Based Learning [Mesh Terms] | | | | |  |  |
|  |  | |  | #13 PBL [Title/Abstract] | | |  |  |  |  |
|  |  | |  | #14 #12 OR #13 | |  |  |  |  |  |
|  |  | |  | #15 Case-based learning [Title/Abstract] | | | |  |  |  |
|  |  | |  | #16 CBL [Title/Abstract] | | |  |  |  |  |
|  |  | |  | #17 #15 OR #16 | |  |  |  |  |  |
|  |  | |  | #18 #5 and #8 and #11 and #14 and #17 | | | | |  |  |
| Inclusion criteria | | |  | Study type: Randomized controlled trial and observational studies. | | | |  |  |  |
|  |  | |  | Subjects: Medical students or trainees in clinical teaching of interventional radiology for hepatocellular carcinoma. | | | | | |  |
|  |  | |  | Intervention measures: The observation group adopted PBL or CBL teaching method or PBL teaching method combined with other teaching methods. | | | | | |  |
|  |  | |  | Evaluation indicators: Including professional theory scores, practical skills scores and questionnaire results. | | | | | |  |
| Exclusion criteria | | |  | Teaching design of non-liver cancer intervention. | | | | | |  |
|  | | |  | Exclusion of guidelines, editorial reviews, case report, case series | | | | | |  |
|  |  | |  | and thesis. | |  |  |  |  |  |
| Selection process | | |  | Tow reviewer selected studies and extracted data independently. | | | | | |  |

**Table S2**

MD and 95% CI for the comparative efficacy of theoretical knowledge examination scores

| PBL+CBL |  |  |  |  |
| --- | --- | --- | --- | --- |
| -1.56 (-3.9, 0.8) | PBL+TBL |  |  |  |
| **2.91 (1.79, 4.04)** | **4.47 (2.02, 6.9)** | CBL |  |  |
| 0.49 (-0.54, 1.52) | 2.05 (-0.12, 4.19) | **-2.42 (-3.61, -1.24)** | PBL |  |
| **5.63 (4.57, 6.69)** | **7.2 (5.09, 9.29)** | **2.72 (1.48, 3.96)** | **5.14 (4.69, 5.6)** | LBL |

**Table S3**

MD and 95% CI for the comparative efficacy of practical skills examination scores

| PBL+CBL |  |  |  |  |
| --- | --- | --- | --- | --- |
| -2.69 (-5.42, 0.03) | PBL+TBL |  |  |  |
| 1.24 (-0.65, 3.13) | **3.94 (0.98, 6.9)** | CBL |  |  |
| -1.18 (-2.53, 0.18) | 1.51 (-0.96, 3.98) | **-2.42 (-4.11, -0.73)** | PBL |  |
| **5.57 (4.24, 6.88)** | **8.25 (5.86, 10.65)** | **4.32 (2.58, 6.08)** | **6.74 (6.15, 7.34)** | LBL |

**Table S4**

LnRR and 95% CI for the comparative efficacy of learning interest

| PBL+CBL |  |  |  |  |
| --- | --- | --- | --- | --- |
| -0.083 (-0.525, 0.389) | PBL+TBL |  |  |  |
| 0.127 (-0.343, 0.621) | 0.209 (-0.453, 0.867) | CBL |  |  |
| 0.061 (-0.394, 0.529) | 0.142 (-0.508, 0.789) | -0.066 (-0.569, 0.426) | PBL |  |
| **0.466 (0.191, 0.864)** | **0.553 (0.286, 0.890)** | 0.348 (-0.227, 0.951) | 0.417 (-0.141, 1.005) | LBL |

**Table S5**

LnRR and 95% CI for the comparative efficacy of learning efficiency

| PBL+CBL |  |  |
| --- | --- | --- |
| -0.295 (-0.899, 0.334) | PBL+TBL |  |
| **0.513 (0.099, 1.034)** | **0.809 (0.459, 1.249)** | LBL |

**Table S6**

LnRR and 95% CI for the comparative efficacy of method satisfaction degree

| PBL+CBL |  |  |  |
| --- | --- | --- | --- |
| -0.001 (-0.447, 0.443) | CBL |  |  |
| -0.001 (-0.452, 0.443) | -0.001 (-0.443, 0.442) | PBL |  |
| 0.159 (-0.304, 0.628) | 0.159 (-0.296, 0.625) | **0.156 (0.046, 0.313)** | LBL |

**Table S7**

LnRR and 95% CI for the comparative efficacy of literature reading ability

| PBL+CBL |  |  |  |
| --- | --- | --- | --- |
| 0.039 (-0.186, 0.278) | CBL |  |  |
| 0.001 (-0.216, 0.216) | -0.039 (-0.277, 0.185) | PBL |  |
| **0.495 (0.178, 0.929)** | **0.457 (0.057, 0.940)** | **0.499 (0.109, 0.972)** | LBL |

**Table S8**

LnRR and 95% CI for the comparative efficacy of Knowledge understanding degree

| PBL+CBL |  |  |
| --- | --- | --- |
| 0.066 (-0.140, 0.294) | CBL |  |
| 0.052 (-0.1526, 0.278) | -0.015 (-0.248, 0.222) | PBL |

**Table S9**

LnRR and 95% CI for the comparative efficacy of clinical practice capacity

| PBL+CBL |  |  |
| --- | --- | --- |
| **0.256 (0.057, 0.507)** | CBL |  |
| **0.247 (0.047, 0.502)** | -0.009 (-0.297, 0.282) | PBL |

**Table S10**

LnRR and 95% CI for the comparative efficacy of clinical thinking capacity.

| PBL+CBL |  |  |  |  |
| --- | --- | --- | --- | --- |
| 0.122 (-0.312, 0.617) | PBL+TBL |  |  |  |
| 0.159 (-0.013, 0.378) | 0.042 (-0.487, 0.523) | CBL |  |  |
| **0.246 (0.045, 0.501)** | 0.128 (-0.410, 0.630) | 0.085 (-0.172, 0.359) | PBL |  |
| **0.451 (0.118, 0.893)** | **0.329 (0.103, 0.606)** | 0.289 (-0.109, 0.763) | 0.204 (-0.215, 0.689) | LBL |
