# Supplementary material for: The effectiveness of problem-based learning and case-based learning teaching methods in clinical practical teaching in TACE treatment for hepatocellular carcinoma in China: a bayesian network meta-analysis
Source: BMC Med Educ. 2024 Jun 17;24:665. doi: 10.1186/s12909-024-05615-8 (PMC11184776; doi:10.1186/s12909-024-05615-8)
Supplement: Supplementary file 2 — Supplementary Material 2 [file 12909_2024_5615_MOESM2_ESM.docx]

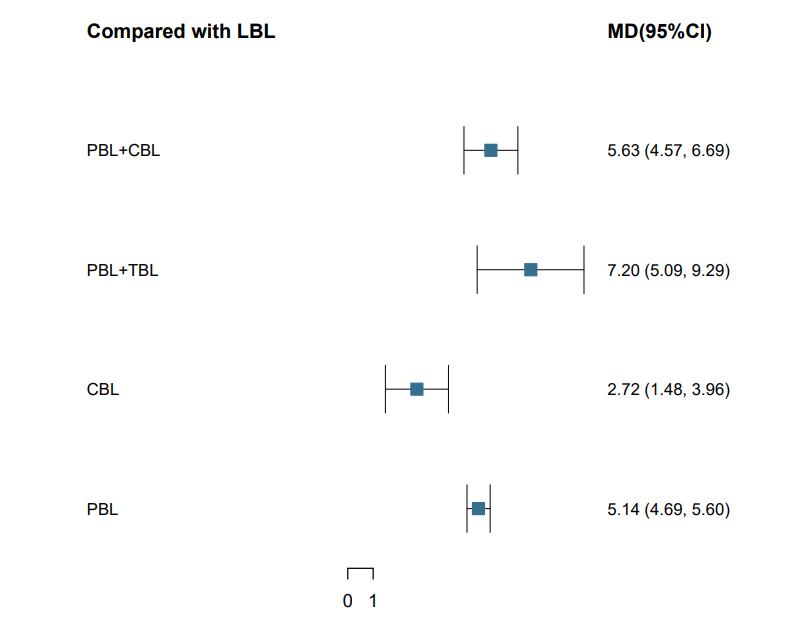


Figure S1 Forest plot of Theoretical knowledge examination scores


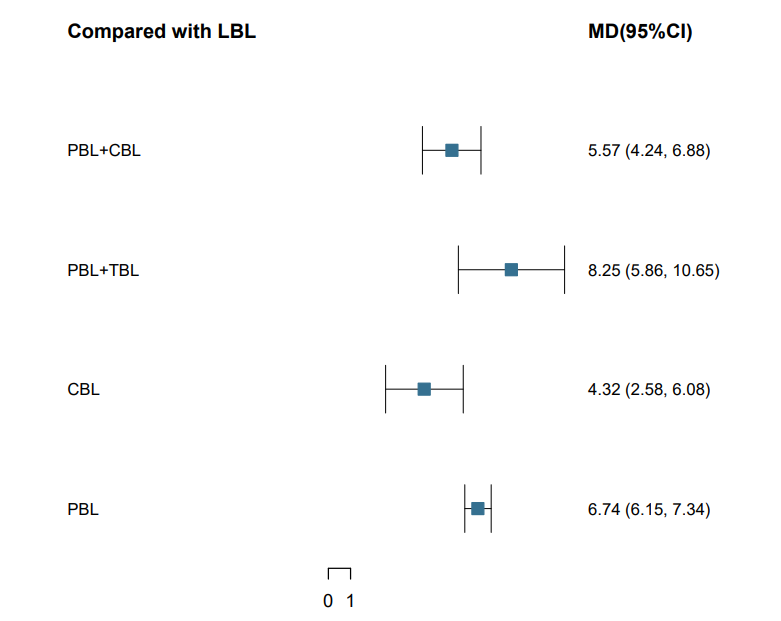


Figure S2 Forest plot of practical skills examination scores


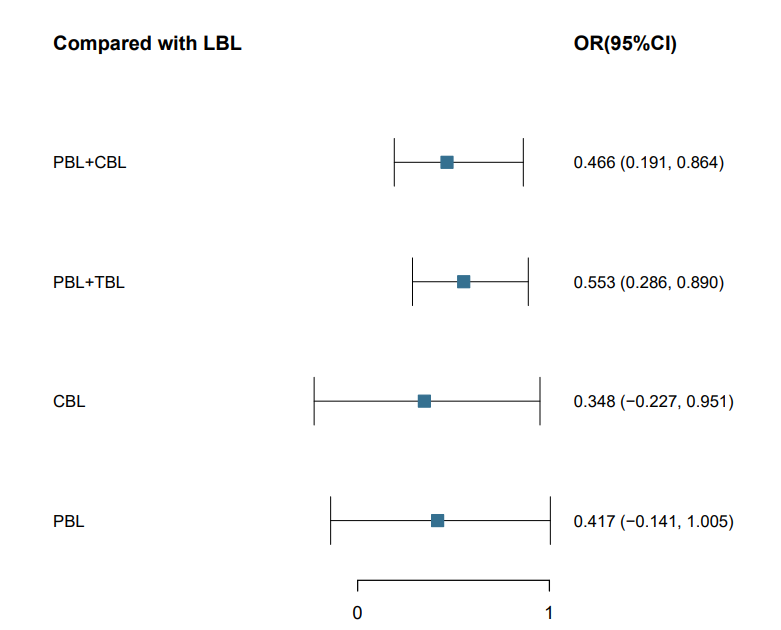


Figure S3 Forest plot of learning interest


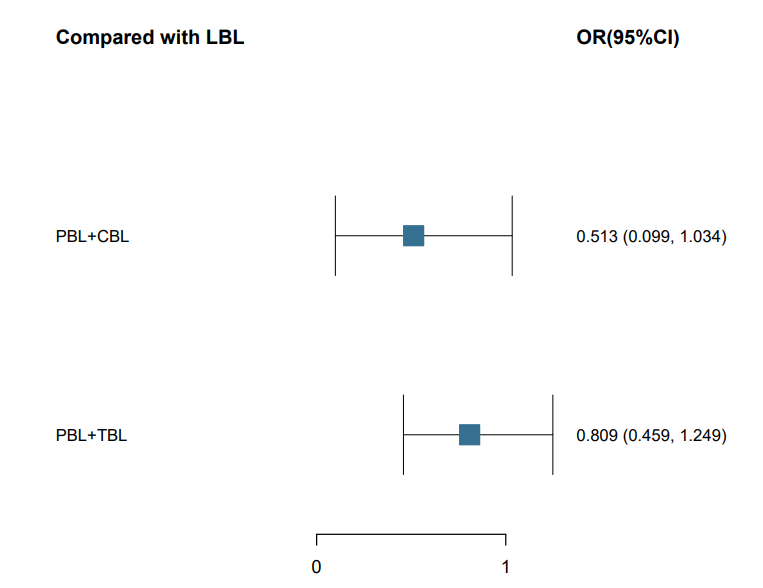


Figure S4 Forest plot of learning efficiency


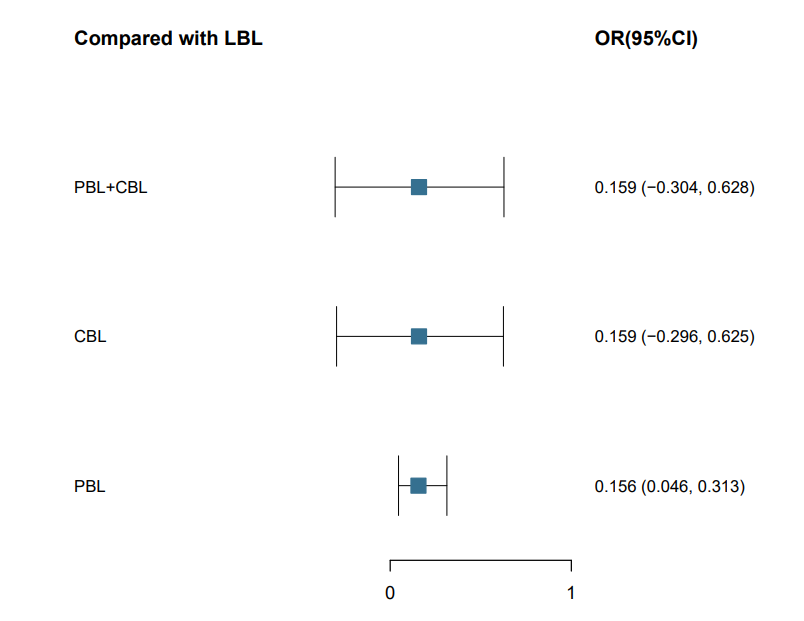


Figure S5 Forest plot of method satisfaction degree


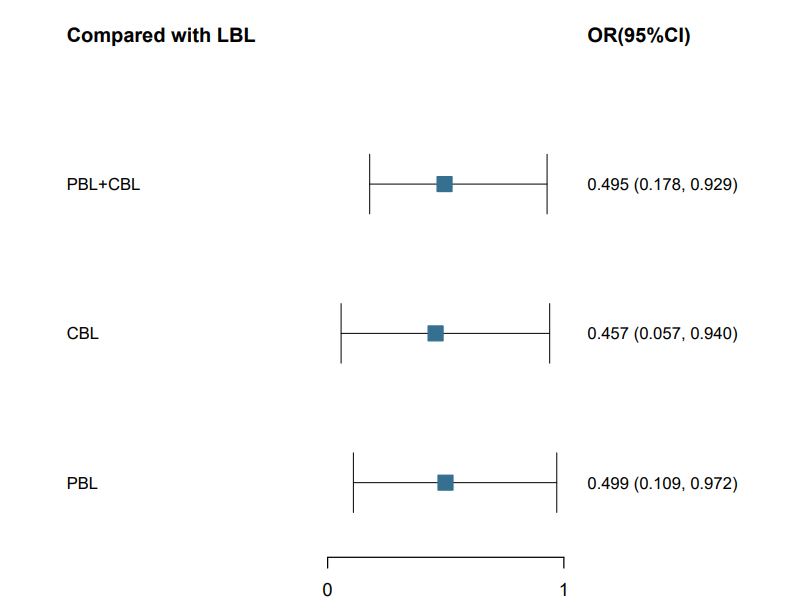


Figure S6 Forest plot of literature reading ability


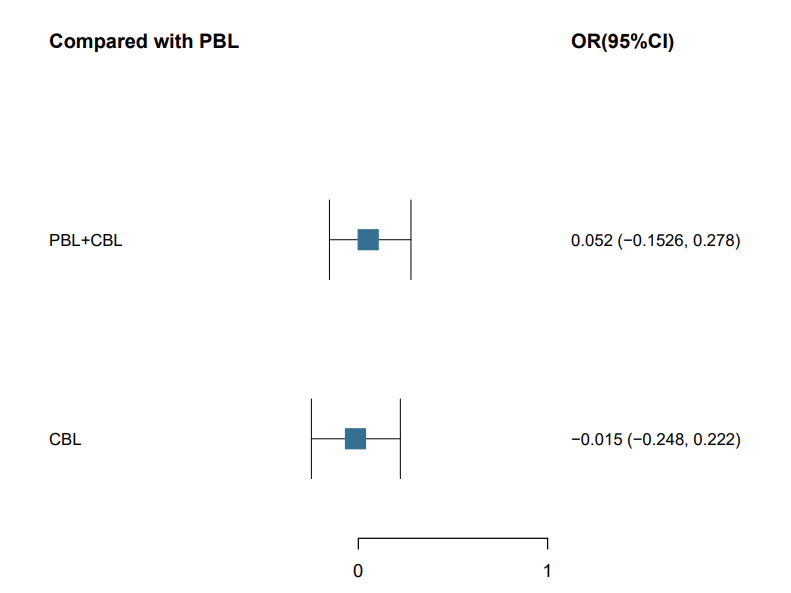


Figure S7 Forest plot of knowledge understanding degree.


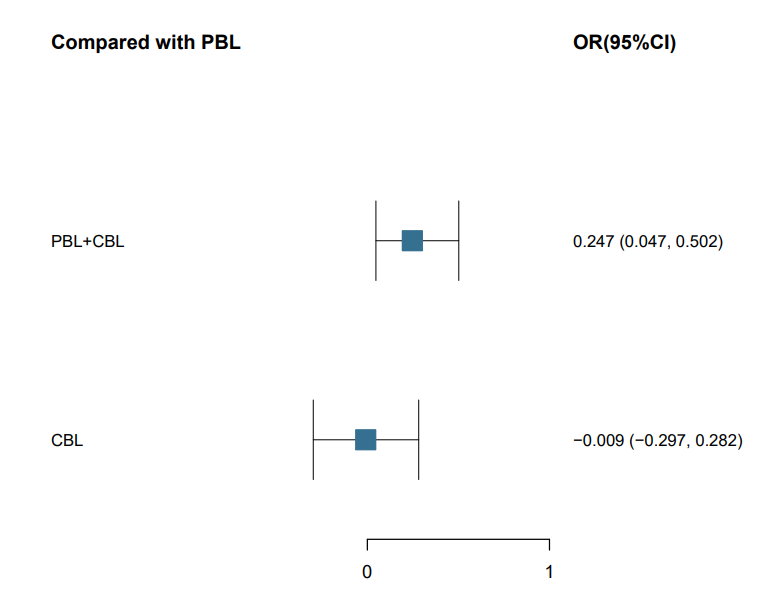


Figure S8 Forest plot of clinical practice capacity


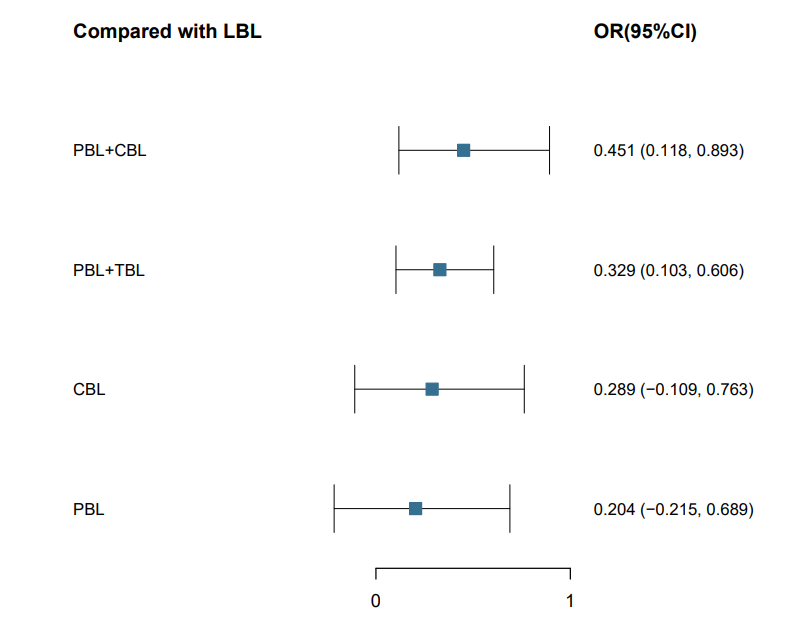


Figure S9 Forest plot of clinical thinking capacity.
